# A discovery system for narrative query graphs: entity-interaction-aware document retrieval

**DOI:** 10.1007/s00799-023-00356-3

**Published:** 2023-04-24

**Authors:** Hermann Kroll, Jan Pirklbauer, Jan-Christoph Kalo, Morris Kunz, Johannes Ruthmann, Wolf-Tilo Balke

**Affiliations:** 1 Institute for Information Systems, TU Braunschweig, Mühlenpfordtstr. 23, 38106 Braunschweig, Lower Saxony Germany; 2 Knowledge Representation and Reasoning Group, VU Amsterdam, De Boelelaan 1111, 1081 HV Amsterdam, The Netherlands

**Keywords:** Narrative information access, Narrative queries, Graph-based retrieval, Digital libraries

## Abstract

Finding relevant publications in the scientific domain can be quite tedious: Accessing large-scale document collections often means to formulate an initial keyword-based query followed by many refinements to retrieve a *sufficiently complete, yet manageable* set of documents to satisfy one’s information need. Since keyword-based search limits researchers to formulating their information needs as a set of unconnected keywords, retrieval systems try to guess each user’s intent. In contrast, distilling short narratives of the searchers’ information needs into simple, yet precise entity-interaction graph patterns provides all information needed for a precise search. As an additional benefit, such graph patterns may also feature variable nodes to flexibly allow for different substitutions of entities taking a specified role. An evaluation over the PubMed document collection quantifies the gains in precision for our novel entity-interaction-aware search. Moreover, we perform expert interviews and a questionnaire to verify the usefulness of our system in practice. This paper extends our previous work by giving a comprehensive overview about the discovery system to realize narrative query graph retrieval.

## Introduction

PubMed, the world’s most extensive digital library for biomedical research, consists of about 34 million publications and is currently growing by more than one million publications each year. Accessing such an extensive collection by simple means such as keyword-based retrieval over publication texts is a challenge for researchers, since they simply cannot read through hundreds of possibly relevant documents, yet cannot afford to miss relevant information in retrieval tasks. Indeed, there is a dire need for retrieval tools tailored to specific information needs in order to solve the above conflict. For such tools, deeper knowledge about the particular task at hand and the specific semantics involved is essential. Taking a closer look at the nature of scientific information search, interactions between entities can be seen to represent a short narrative [15]—a short story of interest: how or why entities interact, in what sequence or roles they occur, and what the result or purpose of their interaction is [6, 15]. This article is an extended version of our previous article [18].

Indeed, an extensive query log analysis on PubMed in [10] clearly shows that researchers in the biomedical domain are often interested in interactions between entities such as drugs, genes, and diseases. Among other results, the authors report that (a) on average significantly more keywords are used in PubMed queries than in typical Web searches, (b) result set sizes reach an average of (rather unmanageable) 14,050 documents, and (c) keyword queries are on average 4.3 times refined and often include more specific information about the keywords’ intended semantic relationships, e.g., *myocardial infarction AND aspirin* may be refined to *myocardial infarction prevention AND aspirin*. Given all these observations, native support for entity-interaction-aware retrieval tasks can be expected to be extremely useful for PubMed information searches and is quite promising to generalize to other kinds of scientific domains, too. However, searching scientific document collections curated by digital libraries for such narratives is tedious when restricted to keyword-based search, since the same narrative can be paraphrased in countless ways [1, 10].

Therefore, we introduce the novel concept of *narrative query graphs for scientific document retrieval* enabling users to formulate their information needs as entity-interaction queries explicitly. Complex interactions between entities can be precisely specified: simple interactions between two entities are expressed by a basic query graph consisting of two nodes and a labeled edge between them. Of course, by adding more edges and entity nodes, these basic graph patterns can be combined to form arbitrarily complex graph patterns to address highly specialized information needs. Moreover, narrative query graphs support *variable nodes* supporting a far broader expressiveness than keyword-based queries. As an example, a researcher might search for treatments of *some disease* using *simvastatin*. While keyword-based searches would broaden the scope of the query far in excess of the user intent by just omitting any specific disease’s name, narrative query graphs can focus the search by using a variable node to find documents that describe treatments of *simvastatin* facilitated by an entity of the type *disease*.

In contrast to query languages for knowledge graphs, our discovery system does not match the query against a single knowledge graph. Instead, we must on-the-fly match the query against several document graphs, i.e., the document itself stays in the focus of the system. And moreover, if variables are used in searches, the result lists require novel visualizations, e.g., clustering document result lists by possible node substitutions to get an entity-centric literature overview. Since our document graphs are extracted from texts with automated methods, we provide provenance information to explain why a document matches the query.

Whereas our previous article [18] focused on benefits of the overall retrieval, this article extends the previous work by describing the extraction workflow in more detail, and the overall discovery system with its key features. In addition, we utilize an Open IE system for a retrieval quality comparison. We also point out limitations that have to be faced in the future to further improve this kind of retrieval. In summary, our contributions are:

1. We proposed narrative query graphs for scientific document retrieval enabling fine-grained modeling of users’ information needs. Moreover, we boosted query expressiveness by introducing variable nodes for document retrieval.
2. We developed a discovery system that processes arbitrary narrative query graphs over the biomedical literature. As a showcase, the service performs searches on 34 million PubMed titles and abstracts in real time.
3. We extended our previous work by stating details on the extraction quality. In addition, we described system details required to implement narrative query graph retrieval.
4. We evaluated our system in two ways: On the one hand, we demonstrated our retrieval system’s usefulness and superiority over keyword-based search on the PubMed digital library in a manual evaluation which included practitioners from the pharmaceutical domain. On the other hand, we performed interviews and a questionnaire with eight biomedical experts who face the search for literature on a daily basis.

## Related work

Relevant research areas to this work are narrative information access, machine learning for retrieval, graph-based retrieval, document representations, and scholarly knowledge graphs.

### Narrative information access

Narrative query graphs are designed to offer complex querying capabilities over scientific document collections aiming at high precision results. Focusing on retrieving entity interactions, they are a subset of our conceptual overlay model for representing narrative information [15]. Our conceptual model narrative allows users to state their information needs as a complex and nested graph model involving entities, events, literals, and even nested literals. We then understand the narrative as a *logical overlay over knowledge repositories*, i.e., we try to find evidence by binding parts of modeled narrative against real-world data. We discussed suitable methods and the technical challenges to bind against document collections in [16]. Here we are looking for scientific narratives that may require combining several statements. We already know that combining statements from different scientific contexts can be a serious threat to the overall result quality [14]. Our proposed discovery system requires that a whole information need must be matched within a small abstract because we assume the context to be stable within it [14, 20].

This work builds upon our previous work [18]. In extension to [18], this paper describes the complete retrieval method and evaluation of narrative query graphs for document retrieval. Therefore, we extend our previous work by giving insights into our data model, corresponding extraction statistics, and the complete extraction workflow. We also utilize an Open IE system for a retrieval quality comparison. In addition, we describe the discovery system in more detail. Mainly, we discuss our design decisions to engage technical challenges. We also show extensions of our original discovery system: A concept selection picker, user feedback options, and Drug Overviews (Drug-centered overviews generated from the literature). We finally point out limitations that have to be faced in the future to improve this kind of retrieval further.

### Machine learning for retrieval

Modern personalized systems try to guess each user’s intent and automatically provide more relevant results by query expansion; see [1] for a good overview. Mohan et al. focus on information retrieval of biomedical texts in PubMed [28]. The authors derive a training and test set by analyzing PubMed query logs and train a deep neural network to improve literature search. Entity-based language models are used to distinguish between a term-based and entity-based search to increase the retrieval quality [33]. Yet, while a variety of approaches to improve result rankings by learning how a query is related to some document  [28, 43, 45] have been proposed, gathering enough training data to effectively train a system for all different kinds of scientific domains seems impossible. Specialized information needs, which are rarely searched, are hardly covered in such models.

### Graph-based retrieval

Using graph-based methods for textual information retrieval gained in popularity recently [6, 35, 36, 45], for instance, Dietz et al. discuss the opportunities of entity linking and relation extraction to enhance query processing for keyword-based systems [6], and Zhao et al. demonstrate the usefulness of graph-based document representations for precise biomedical literature retrieval [45]. Kadry et al. also include entity and relationship information from the text as a learning-to-rank task to improve support passage retrieval [12]. Besides, Spitz and Gertz built a graph representation for Wikipedia to answer queries about events and entities more precisely [35]. But in contrast to our work, those approaches focus on unlabeled graphs or include relationships only partially.

### Document representation

Croft et al. proposed a network representation of documents and their corresponding terms [5]. Such a network representation supports effective retrieval because documents and terms can easily be linked and traversed in the retrieval phase. Further, [4] demonstrated that using a network representation can enhance the effectiveness of a retrieval system while allowing the implementation of several search strategies.

France has developed the MARIAN system that allows an effective representation and retrieval of relationships between digital library objects [9], e.g., how library objects are linked. Another example of an early intelligent retrieval system was the CODER system [38]. The system was implemented in a modular fashion allowing to test novel retrieval strategies. Chen has developed an object-oriented model called LEND (Large External object-oriented Network Database) model [3]. This model supports the representation and querying of graph-structured data.

While the research on effective document representations for retrieval has a long-standing tradition and is still ongoing, the previous works focused on retrieving documents based either on textual content or metadata. In contrast, our work is focused on the representation of documents as entity-interaction-aware graphs, i.e., we break down document texts into graphs.

### Scholarly knowledge bases

Several projects aim to capture knowledge about the academic world as graph representations, e.g., the Microsoft Academic Knowledge Graph [8], the Open Research Knowledge Graph [11], and OpenAlex [31]. Another example is GrapAl, a graph database of academic literature that is designed to assist academic literature search by supporting a structured querying language, namely Cypher [2]. GrapAl mainly consists of traditional metadata like authors, citations, and publication information but also includes entities and relationship mentions. However, complex entity interactions are not supported, as only a few basic relationships per paper are annotated.

QKBfly is a search system that extracts facts from text to support question answering [29]. It constructs a knowledge base for ad hoc question answering during query time that provides journalists with the latest information about emergent topics. However, they focus on retrieving relevant facts concerning a single entity. In contrast, we focus on document retrieval for complex entity interactions, i.e., we match structured queries against documents to retain the original contexts.

In contrast to the previous works, this paper introduces a complete discovery system involving extraction, retrieval, user interface design, effectiveness evaluation, and user studies.

## Narrative query graphs

Entities represent things of interest in a specific domain: drugs and diseases are prime examples in the biomedical domain. An entity $e = (ID, type)$, where *id* is a unique identifier and *type* the entity type. To give an example, we may represent the drug *simvastatin* by its identifier and entity type as follows: $e_{\textit{simvastatin}}= ( D019821 , Drug )$. Typically, entities are defined by predefined ontologies, taxonomies, or controlled vocabularies, such as NLM’s MeSH or EMBL’s ChEBI. We denote the set of known entities as $\mathcal {E}$. Since we aim to find entity interactions in texts, we need to know where entities are mentioned. In typical natural language processing, each sentence is represented as a sequence of tokens, i.e., single words. Therefore, an **entity alignment** maps a token or a sequence of tokens to an entity from $\mathcal E$ if the tokens refer to it.

Entities might also be classes as well, e.g., the entity *diabetes mellitus* (Disease) refers to a class of specialized diabetes diseases such as *DM type 1* and *DM type 2*. Thus, these classes can be arranged in subclass relations, i.e., *DM type 1* is the subclass of general *diabetes mellitus*. We define the following function to derive the set of all subclasses of an entity: $\textit{subclasses}(e) = \lbrace e_i \mid e_i \textit{ is subclass of e}\rbrace$. If an entity *e* is not a class or does not have any subclasses, the function does simply return *e*.

We call an interaction between two entities a **statement** following the idea of knowledge representation in the Resource Description Framework (RDF) [26]. Hence, we define a **statement** as triple (*s*, *p*, *o*) where $s,o \in \mathcal {E}$ and $p \in \Sigma$. $\Sigma$ represents the set of all interactions we are interested in. We focus only on interactions between entities, unlike RDF, where objects might be literals too. For example, a *treatment* interaction between *simvastatin* and *hypercholesterolemia* is encoded as ($e_{\textit{simvastatin}}$, *treats*, $e_{\textit{hypercholesterolemia}}$). We call a set of extractions from a single document a **document graph**.

Document graphs support narrative querying, i.e., the query is answered by matching the query against the document’s graph. Suppose a user formulates a query like ($$e_{\textit{simvastatin}}$$, *treats*, $$e_{\textit{hypercholesterolemia}}$$). In that case, our system retrieves a set of documents containing the searched statement. Narrative query graphs may include typed variable nodes as well. A user might query ($$e_{\textit{simvastatin}}$$, *treats*, *?X(Disease)*), asking for documents containing *some* disease treatment with *simvastatin*. Hence, all documents that include *simvastatin* treatments for diseases are proper matches. Formally, we denote the set of all variable nodes as $$\mathcal {V}$$. Variable nodes consist of a name and an entity type to support querying for entity types. We also support the entity type *All* to query for arbitrary entities. We write variable nodes by a leading question mark. Hence, a narrative query graph might include entities stemming from $$\mathcal {E}$$ and variable nodes from $$\mathcal {V}$$. Formally, a **fact pattern** is a triple $$ fp = (s, p, o)$$ where $$s, o \in (\mathcal {E} \cup \mathcal {V})$$ and $$p \in \Sigma $$. A **narrative query graph** *q* is a set of fact patterns similar to SPARQL’s basic graph patterns [30]. When executed, the query produces one or more matches $$\mu $$ by binding the variable symbols to actual entities, i.e., $$\mu : \mathcal {V} \rightarrow \mathcal {E}$$ is a partial function. If several fact patterns are queried, all patterns must be contained within a document forming a proper query answer. Suppose queries include entities that are classes and have subclasses. In that case, the query will be expanded to also query for these subclasses, i.e., direct and transitive subclasses. We do this by applying the $$\textit{subclasses}$$ function on every entity in the query.

## Document graphs

The discovery system requires a transformation of documents’ texts into a document graph representation. This step involves entity linking, information extraction, cleaning, and loading. It extracts document graphs from text and stores them in a structured repository. Then the system takes narrative query graphs as its input and performs graph pattern matching. All document graphs that match the query are returned to the users. In this section, we describe all relevant details about the extraction process.

### Document graph extraction

Linking entities and extracting statements from texts form the essential core of mining document graphs. Therefore, we analyzed a plethora of different domain-specific methods like supervised annotations tools (e.g., TaggerOne [24] and GNormPlus [40]). For the extraction phase, we analyzed supervised extraction tools that aim to reduce the need for training data (e.g., Snorkel [32] and DeepDive [34]). However, all of these supervised methods still require training data and are thus specialized for a certain domain. Although their quality is often very high, we went for a different approach: unsupervised linking and extraction. Our goal was to design and utilize methods that deliver sufficient quality and could still be transferred to another domain. With such a set of methods realizing the service in a different domain seems not too far-fetched.

Our efforts yielded a toolbox that we shared as open source^1^: *A Toolbox for the Nearly-Unsupervised Construction of Digital Library Knowledge Graphs* [17]. The toolbox includes methods for unsupervised entity linking, interfaces to unsupervised extraction methods, and cleaning methods to obtain a sufficient quality. We call it nearly unsupervised because the toolbox requires the design of two different vocabularies: (1) An entity vocabulary including all entities of interest. Each entry consists of an unique entity id, an entity type, an entity name, and a list of synonyms. (2) A relation vocabulary including all relations of interest. Each entry consists of a relation and a set of synonyms. For details about the actual extraction quality, we refer the reader to our original toolbox paper for a quantitative evaluation in the biomedical domain [17] and our follow-up work on a qualitative analysis for three corpora: pharmaceutical literature, the Wikipedia encyclopedia, and political sciences literature [19]. In brief, our main findings were: First, entity and relation vocabularies are a fixed requirement to apply the toolbox. Second, the quality clearly lags behind supervised entity linking and information extraction methods. Third, the canonicalization of verb phrases to precise relations is still an open issue in some cases. Although missing vocabularies, limited extraction quality (especially recall), and open canonicalization issues must be tackled in the future, we still argue that nearly unsupervised workflows are worth studying in digital libraries because they completely bypass training data in the extraction phase [19].

**Table 1:** Number of entries in our entity vocabulary

Entity type	#Distinct entries	#Terms
Chemical	146	1,850
Disease	5051	57,295
Drug	45,200	69,767
Dosage form	136	6,891
Excipient	12,951	132,704
Lab method	528	5,742
Method	2,512	23,182
Plant family	2,818	2,818
Vaccines	161	1032
Sum	69,503	300,134

**Table 2:** Evaluation of our entity linking step: linking of chemicals and diseases was tested on two established biomedical benchmarks (BioCreative V CD-R [41] and NCBI disease [7]). For drugs, dosage forms, excipients, and plant families, we performed a manual evaluation of 50 random-sampled annotations

Entity type	Benchmark	Precision	Recall	F1
Chemical	BioCreative V CD-R	76.6%	78.7%	77.6%
Disease	BioCreative V CD-R	82.8%	62.0%	70.9%
Disease	NCBI disease	74.5%	55.1%	63.3%
Drug	Sample	90.0%	–	–
Excipient	Sample	74.0%	–	–
Dosage form	Sample	82.0%	–	–
Plant family	Sample	82.0%	–	–

### Pharmaceutical entity linking

For our retrieval system we designed an entity vocabulary that comprises *chemicals, drugs, diseases, dosage forms, excipients, plant families, lab methods, methods, and vaccines*. We derived vocabulary entries from the biomedical specialized database ChEMBL [27], the Medical Subject Headings (MeSH),^2^ and Wikidata [37]. In cooperation with two pharmaceutical domain experts, we manually selected suitable subsets of the previous vocabularies and manually formulated missing entities such as specialized dosage forms (e.g., nanoparticles). In summary, we derived 69,503 distinct entities with 300,134 terms. A list of all entity types and their corresponding vocabulary size is shown in Table 1.

We then evaluated our entity linking quality for our work  [17]. For chemicals and diseases, we selected two biomedical benchmarks: BioCreative V CD-R and NCBI Disease. We used the given vocabularies for these benchmarks and applied our dictionary-based entity linker. In addition, we randomly sampled 50 entity annotations for drug, dosage forms, and plant families. We presented these annotations to two pharmaceutical domain experts. Together they decided for each text span if it was linked correctly to the given entity. However, we could thus only compute the precision for these three entity types. The results are shown in Table 2.

Diseases could be linked with a precision between 55.1 and 82.8%. The recall was between 62.0 and 63.3%. For chemicals, we obtained a precision of 76.6% and 78.7%. In our sample-based evaluation, we obtained a precision of 90% for drugs, 82% for dosage forms and plant families, and 74% for excipients. We evaluated the linker against state-of-the-art supervised methods in a previous publication; see [17] for more details.

To give a few more insights: We decided not to link trade names of drugs. These trade names included words like *horse*, *man*, *power*, etc., which were often linked incorrectly. For our discovery system, the main issue was that a few frequently but wrongly linked entities would be annoying for users to handle. We handled this issue by applying two strategies:

1. We went through the 500-top-frequently tagged entities and removed often wrongly linked entities from our vocabularies.
2. We applied a special cleaning rule for plant families like *paris* because they were of high interest for our purposes but often linked wrongly.

We checked whether one of 82 regular expressions (e.g., *Traditional Medicine* or phytotherap*) could be matched against the same abstract. We kept the linked plant families only if at least one of these expressions could be successfully matched. In summary, dictionary-based entity linkers do have their limitations. But we did not need training data for the linking step and the quality was sufficient for us to continue.

In addition to our entity linking workflow, we integrated annotations from the PubTator Central service.^3^ This service is hosted by the National Library of Medicine (NLM) and allowed us to retrieve annotations for diseases, chemicals, genes, and species. We analyzed the annotation service in cooperation with our domain experts. For our goal, we found the chemical annotations to be too general. That is why we integrated only diseases, genes, and species annotations. Details about PubTator Central can be found in [39, 42].

### Pharmaceutical inf. extraction

We had to extract statements between the detected entities for the actual document graph representation. Although supervised extraction methods would have likely achieved a better extraction quality, we decided to build upon unsupervised extraction methods. The quality of existing open information extraction like OpenIE 6 sounded promising [13], but we found that open information extraction methods highly lack recall when processing biomedical texts; see the evaluation in [17]. That is why we developed a recall-oriented extraction technique **PathIE** in [17] that flexibly extracts interactions between entities via a path-based method. The central idea was to take sentences in which at least two different entities have been detected. Then, the shortest path between the entities in the grammatical structure of the sentence was computed. All verb phrases and keywords (that have been specified in the relation vocabulary) were considered for extraction. PathIE then yielded triples consisting of two entities and a predicate (either a verb phrase or a given keyword like treatment).

PathIE yielded many synonymous predicates (treats, aids, prevents, etc.) that represent the relation *treats*. The toolbox implemented a canonicalization procedure to unify synonymous predicates to precise relations. The procedure works as follows: Given a pre-designed relation vocabulary, all terms that appear directly in the vocabulary are mapped to the corresponding relation. In addition, we used the optional word embedding feature to also canonicalize similar verb phrases, i.e., verb phrases that were similar to entries in the vocabulary were also mapped to the corresponding relation.

The pharmaceutical relation vocabulary had to have precise semantics and was built with the help of two domain experts. The relation vocabulary included 60 entries (10 relations plus 50 synonyms) for the cleaning step. As a biomedical word embedding, we used the pre-trained word embedding from [44]. Then, we applied the toolbox canonicalization procedure. The cleaning allowed users to formulate their queries based on a well-curated vocabulary of entity interactions in the domain of interest. To increase the quality of extractions, we introduced type constraints by providing fixed domain and range types for each interaction. Extracted interactions that did not meet the interaction’s type constraints were removed. For example, the interaction *treats* is typed, i.e., the subject must be a drug, and the object must be a disease or species. Some interactions in our vocabulary like *induces* or *associated* are more general and thus were not annotated with type constraints. We found those type constraints worked well if the relations are directed, e.g., a *treats* relation between a drug and a disease [19]. If relations are not directed, PathIE often messes up the direction by design, e.g., a causes b instead of b causes a.

**Table 3:** CDR2015 benchmark evaluation [41]. The table reports the extraction quality for CoreNLP OpenIE, PathIE, and best reported baselines

Method	Prec. (%)	Rec. (%)	F1 (%)
CoreNLP OpenIE	64.9	5.8	10.6
PathIE	50.8	31.7	39.1
Best precision	90.5	80.8	85.4
Best recall	86.1	86.2	86.1

The following experiment has already been reported in [17]. To test our extraction pipeline, we utilized the benchmark of [41]. The benchmark provides abstracts and entity annotations and requires extracting *induce* relations between chemicals and diseases. We loaded the abstracts and annotations. Then, we applied PathIE and the canonicalization with our relation vocabulary. For comparison, we applied the Stanford CoreNLP method [25] from our toolbox. The results are reported in Table 3. The table lists the precision, recall and F1 score when extracting statements with our toolbox using either CoreNLP OpenIE or PathIE. In addition, we included the workshop’s best-performing systems [41] concerning precision and recall in Table 3.

In brief, PathIE achieved an F1 score of 39.1%, whereas supervised methods achieved an F1 score between 85.4% and 86.1%. CoreNLP achieved a precision of 64.9% but a recall of only 5.8%. Due to the low recall, we decided to use PathIE for our retrieval system. However, we report on another comparison between PathIE and CoreNLP for the actual retrieval in our evaluation section. We refer the reader for more details about the toolbox’s extraction quality to our previous publications [17, 19].

**Table 4:** Number of extracted statements per relation

Relation	#Statements
Associated	182,258,817
Method	153,040,391
Compares	19,552,314
Induces	12,012,245
Treats	9,938,585
Administered	7,098,069
Decreases	4,299,302
Interacts	4,002,765
Inhibits	1,741,788
Metabolizes	112,822
Sum	394,057,098

**Fig. 1:**
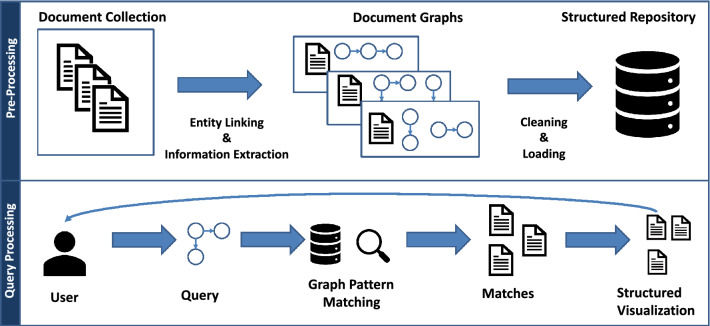
System overview: document graphs are extracted from texts, cleaned, indexed, and loaded into a structured repository. Narrative query graphs are then matched against this repository to retrieve the respective documents

For our service, we applied three special rules:

1. Instead of removing statements, we mapped all statements that hurt their type constraints to the *associated* relation since both entities were still in *some way* associated in the sentence.
2. All statements including an entity of type *method* or *lab method* were mapped to the relation *method*.
3. All statements including an entity of type *dosage form* were mapped to the relation *administered*.

We applied the first rule to allow users to search for *arbitrary* relations between entities. Rules 2 and 3 were applied to have special relations for methods and dosage forms. In summary, we extracted 394 M statements and ten unique relations. Statistics are reported in Table 4.

## Discovery system

In the following section, we describe our discovery system for entity-interaction-aware document retrieval. On the one hand, the system must be capable of answering narrative query graphs. On the other hand, querying in this way requires suitable interfaces for a suitable user experience. Moreover, our discovery allows users to integrate variables into their searches which asks for novel visualization in the user interface. Our discovery system is freely accessible.^4^ A systematic overview of the whole system is depicted in Fig. 1. In the following, we report on the current system’s version (July 2022).

### Discovery content

We integrated the complete NLM Medline collection (about 34 M publications), i.e., the content of the PubMed search engine. Therefore, we obtained the titles and abstracts plus entity annotations from the PubTator service. In addition, we loaded metadata for the publications, e.g., authors, journal, publication year, etc. We obtained the metadata from the NLM’s official XML dumps. In joint cooperation with ZB MED and the Robert Koch-Institute in Germany, we integrated about 45k pre-prints from PreView [22, 23] (ZB MED service) for COVID-19 questions. In both cases, we loaded each publication’s titles, abstracts, and metadata (authors, journal, etc.). We did not consider full texts. We then applied our entity linking, information extraction, and cleaning workflow to transform each document’s text into a document graph. Note that we concatenated a document’s title and abstract to derive a single text for the graph transformation. In addition, we could not extract a document graph for all documents since we neither detected entities nor interactions in them. For instance, we extracted *19.5 M* graphs from *34 M* PubMed documents and *24.6k* from *45k* COVID-19 pre-prints. The statistics are reported in Table 5. We developed scripts to update the service content at periodic intervals. At the moment, the discovery system cannot search for documents from which we cannot extract a single statement. In the future, a more flexible query model could allow a search for that documents, i.e., by rewriting a narrative query graph to a set of keywords if no match could be found otherwise.

**Table 5:** Statistics about the content of our system

Name	#Docs	#Graphs
PubMed	34 M	19.5 M
COVID-19 Pre-Prints	45k	24.6k

**Fig. 2:**
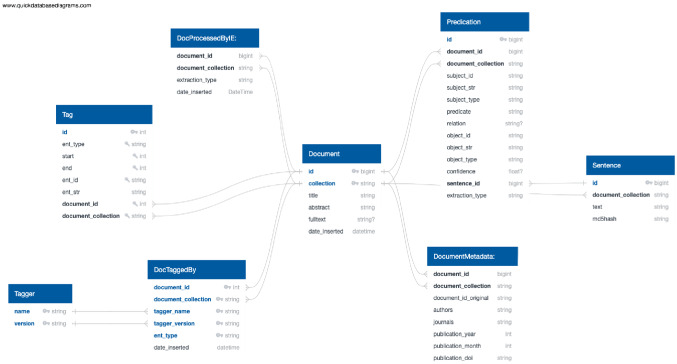
Database schema overview: document is the central table storing titles and abstracts. Tables on the left side store information about the entity linking. Tables on the right side store information about the extraction process. The document metadata stores information for the service

### Data representation

In the design of our discovery system, we had two central requirements: (1) process narrative query graphs and (2) deliver a suitable user experience. In early talks with domain experts, we found that explainability was relevant, i.e., visualizing why documents should match their information needs. With that in mind, the question of how we should represent our data was raised. In an early phase, we decided to store our data in a relational database because they are well supported (reliable software and interfaces) and our data could be broken in a relational fashion. For example, the service returns document titles, sentences, entity annotations, and extraction information to explain matches to the user. In this way a central document table allowed us to join the corresponding information if necessary. An overview of our relational schema is shown in Fig. 2.

The document table stores the title and abstract for each document. Each document is identified by an ID and the corresponding collection (e.g., PubMed). The tag table stores entity annotations, the predication table stores the extracted statement, and the document metadata tables stores information about a publication’s authors, journals, etc. To explain matches to the user, we integrated a sentence table to link an extracted statement to its sentence origin. We split sentences and statements to reduce redundancy - several statements might have been extracted from the same sentences. Sentences are identified by an MD5-hash for each document collection. To accelerate the actual retrieval of documents’ metadata, we created a materialized view *metadata service* which contains titles and metadata of documents in which at least a single statement was extracted. On the one hand, titles and metadata can, in this way, be queried from the same table. On the other hand, the number of documents is reduced from 34.4 to 19.5 M entries. In other words, we did not extract a single statement in around 15 M documents. Some database statistics (July 2022) like the number of tuples and size on disk of relevant tables are reported in Table 6. We used Postgres V10 as a relational database implementation. The database (incl. indexes and materialized views) consumed roughly 300GB of disk space in sum.

### Document retrieval

As a reminder of Sect. 3, a narrative query graph consists of fact patterns following simple RDF-style basic graph patterns. Our discovery system automatically translates these narrative query graphs into a structured query language: They are translated into SQL statements for querying the underlying relational database. A single fact pattern requires a selection of the extraction table with suitable conditions to check the entities and the interaction. Multiple fact patterns require self-joining of the extraction table and adding document conditions in the where clause, i.e., the facts matched against the query must be extracted from the same document. In practice, joining the predication table with itself was not fast enough when many rows were selected to answer a fact pattern.

That is why we computed an inverted index. The inverted index mapped subject-predicate-object tuples to a denormalized attribute: This attribute then stored a document ID plus the predication IDs in a JSON format, e.g., the document IDs *1* and the predication IDs *2* and *3*. The predication IDs were required to explain matches to users, i.e., which sentence and why the sentence matched the fact pattern. For subjects and objects we used two attributes: the corresponding entity ID and entity type. The type was helpful to accelerate queries with variables that search for a specific entity type (e.g., drug or disease). We created indexes for the subject, predicate, and object attributes. The inverted index had 34 million tuples and consumed 14 GB of disk space (incl. indexes). Having that index, a fact pattern required only a single selection on it. We developed an in-memory and hash-based matching algorithm that quickly combines the results.

Another issue to think about were ontological subclass relations between entities. For example, querying for treatments of *Diabetes Mellitus* would require to also search for the subclasses *Diabetes Mellitus Type 1* and *Diabetes Mellitus Type 2*. Query rewriting was necessary to compute complete results for queries that involve entities with subclasses [21]. Therefore, we utilized the Medical Subject Headings (MeSH) Ontology and the Anatomical Therapeutic Chemical Classification System (ATC). ATC was used to support querying for classes of drugs. We rewrote queries that include entities with subclasses to also query for these subclasses. If an entity was also a superclass, then we also searched for all subclasses. We rewrote the SQL statement precisely in the following way: Instead of searching for a single entity, we searched with an *IN* expression. We allowed all subclasses plus the given entity.

**Table 6:** Database statistics (July 2022) of our underlying relational database. We report the consumed disk space of relevant database tables (size reflects the pure data while size* includes indexes as well)

Table name	#Tuples	Size	Size*
Document	34.4 M	32 GB	35 GB
Document metadata	34.4 M	8.1 GB	10 GB
Metadata service	19.5 M	6.6 GB	7.2 GB
Tag	524.2 M	44 GB	91 GB
Predication	394 M	61 GB	95 GB
Sentence	67.3 M	17 GB	19 GB

In brief, the query translation works as follows:

1. The user inputs a string through the query builder in the form of a list of subject–predicate–object tuples.
2. We translate each subject and object to a set of corresponding entities, i.e., all entities that have the given term (subject/object) as one of its synonyms.
3. We expand each entity by all subclasses, i.e., we apply the $\textit{subclass}$ function to each entity. The intermediate query representation is now a list of fact patterns. A fact pattern is a triple consisting of a set of entities as the subject, a predicate, and a set of entities as the object.
4. We translate each fact pattern into a SQL statement:

If a subject/object is only a single entity, we directly add a simple comparison in the WHERE clause. If a subject/object is a set of entities, we add an *IN* statement to check whether the entity is in that set of entities. To accelerate the translation, we maintained an in-memory index mapping terms to a set of entities, including all of their subclasses if applicable.

Due to the long-standing development of databases, querying our index was performed very quickly by suitable indexes. Besides, we implemented some optimization strategies to accelerate the query processing, e.g., matching fact patterns with concrete entities first and fact patterns with variable nodes afterward. We remark on our system’s query performance in our evaluation.

#### Remarks

*Why did we not build upon graph databases?* We thought about utilizing graph databases for the query processing. Our main motivation to stick to relational databases was for simplicity reasons: On the one hand, we were familiar with relational architectures. On the other hand, we had to store data about the documents and the extraction information. This way we can identify new documents that must be processed, update suitable tables and indexes, and so on. In addition, the service had to return document and provenance information to the user, i.e., titles, journal, author, and why documents match a user query. For simplicity, we decided to store all data in a single database and only maintain a single one. Moreover, the overall query performance was sufficient for us. For future work, analyzing graph databases like the RDF database Virtuoso could be of interest for our discovery system.

### Architecture

Our service was realized as a web service split into two components: a backend for the query processing and a frontend for user inputs. We implemented the backend as a REST service based on the Django framework in Python. The frontend was implemented by utilizing the Bootstrap and the jQuery Framework. The data exchange between both sides was realized with JSON.

Transferring the results between the backend and frontend turned out to be a challenge. Search engines typically only transfer parts of the result lists. If users move to the next result page, these page results are transferred to the frontend. The problem for us was that we did not have typical result lists in every case. For instance, results for searches with variables had to be aggregated on the backend side before transmission. In brief, a *simple* result list in our system might be composed of several nested lists, i.e., documents that share the same variable substitution. Thus, implementing a *lazy loading* for the next pages was challenging. This feature would have required a complex caching architecture in the backend, i.e., store the result lists and allow the frontend to load specific parts dynamically. An alternative would have been to recompute the same query with corresponding page/list positions. Both options were not suitable for us because they would have consumed too many resources in the backend.

That is why we decided to transfer the complete result object once between the backend and frontend. The JSON contained the basic structure (simple list or nested list), all document information (ID, title, authors, journals, etc.), and provenance information. For provenance information we only transferred IDs of the predication table, i.e., we dynamically loaded provenance information from the backend if the user asked for it. The frontend then dynamically visualizes parts of the results and allows the user to jump in the lists. Depending on the number of results and the usage of variables, the result size can vary between a few KB up to several MB. Very large queries like *Drug treats Disease* would even require to transfer of a few hundred MBs. But the vast majority of queries that contain at least a single entity (and not only variables) require at maximum a few MBs. The Django framework supports sending the data in a compressed format if the browser supports it. We enabled that option to decrease the transmission size. However, we are aware that the transmission size is an issue in practice.

**Fig. 3:**
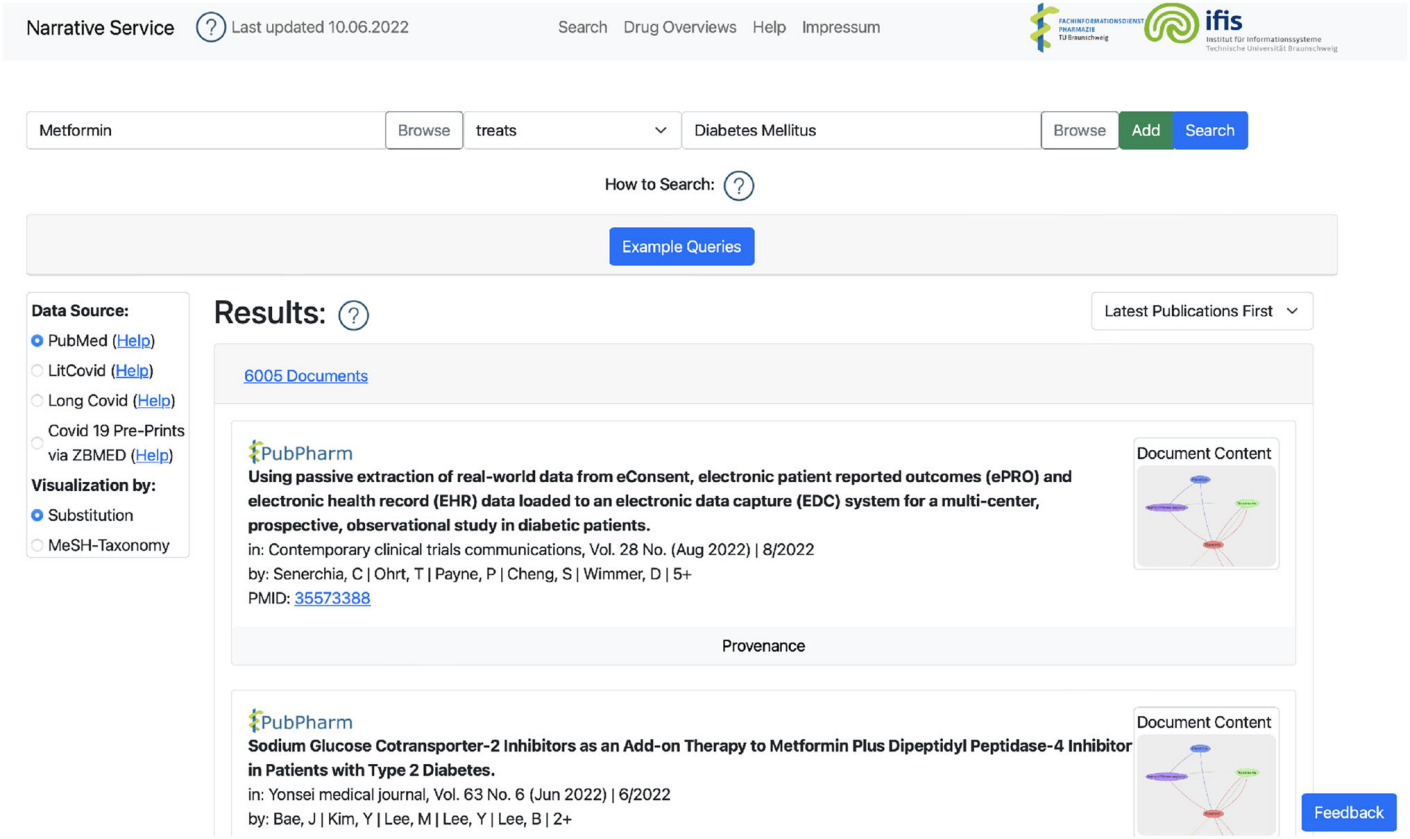
A screenshot of our user interface: The query builder is shown on the top. Users can formulate their queries by adding more patterns and then start their search. On the left side, several filter options are shown. In the center/bottom, the result list is visualized. Each result is represented by metadata and a Provenance button to explain the match

### User interface

In the following, we present a user interface resulting from joint efforts by the University Library, the Institute for Information Systems, and two pharmaceutical domain experts who gave us helpful feedback and recommendations. In contrast to graph query interfaces for SPARQL queries, we wanted to create a user interface that is easy to use and does not require to learn an additional query language. Furthermore, we supported the user with a query builder and suitable result visualization on the frontend side. In an early prototype phase, we tested different user interfaces to formulate narrative query graphs, namely

1. A simple text field,
2. A structured query builder, and
3. A graph designer tool.

We found that our users preferred the structured query builder, which allows them to formulate a query by building a list of fact patterns. For each fact pattern, the users had to enter the query’s subject and object. The service assists the user by suggesting about three million terms (entity names plus synonyms). Then, they could select an interaction between both in a predefined selection. Variable nodes could be formulated, e.g., by writing *?X(Drug)* or just entering the entity-type like *Drug* in the subject or object field.

When users start their search, the service sends the query to the backend and visualizes the returned results. The returned results are sorted by their corresponding publication date in descending order. The service represents documents by a document ID (e.g., PubMedID), a title, a link to the digital library entry, metadata (authors, journal, etc.), and provenance information. Provenance includes the sentence from which the matching fact was extracted. We highlight the linked entities (subject and object) and their interaction (text term plus mapping to the interaction vocabulary). Provenance may be helpful for users to understand why a document is a match. If a query contains multiple fact patterns, we attach a list of matched sentences in the visualization. Visualizing document lists is comparable to traditional search engines, but handling queries with variable nodes requires novel interfaces. In the next subsection, we will discuss such visualizations for queries including variable nodes. A screenshot of the running system is shown in Fig. 3

### Retrieval with variable nodes

**Fig. 4:**
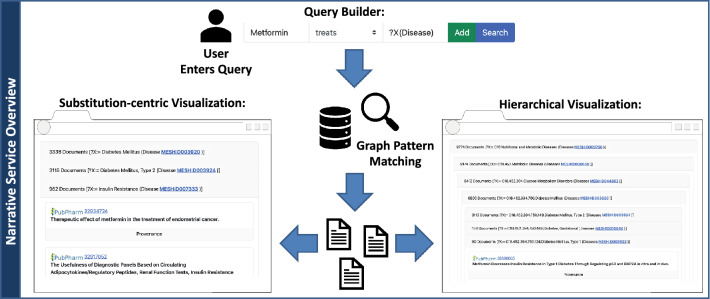
A schematic overview of our service implementation. A query builder helps the users to formulate their information needs. If the narrative query involves variable nodes, the results can be visualized in a substitution-centric visualization (left side) or in a hierarchical visualization (right side)

Variable nodes in narrative query graphs may be restricted to specific entity types like *Disease*. We also allowed a general type *All* to support querying for arbitrary entities. For example, a user might formulate the query (*Simvastatin*, *treats*, *?X(Disease)*). Several document graphs might match the query with different variable substitutions for ?*X*. A document $d_1$ with the substitution $\mu _1(?X)=$ *hypercholestorelemia* as well as a document $d_2$ with $\mu _2(?X)=$ *hyperlipidemia* might be proper matches to the query. How should we handle and present these substitutions to the users? Discussions with domain experts led to the conclusion that aggregating documents by their substitution seems most promising. Further, we present two strategies to visualize these document result groups in a user interface: *substitution-centric* and *hierarchical visualization*. A general overview of both visualizations is shown in Fig. 4. We implemented the aggregation and ranking on the backend side: The frontend sends the selected visualization to the backend. The backend then calculates the required data representation and sends it to the frontend. The frontend finally visualizes the computed representation.

*Substitution-centric visualization.* Given a query with a variable node, the first strategy is to aggregate by similar variable substitutions. We retrieve a list of documents with corresponding variable substitutions from the respective document graphs. Different substitutions represent different groups of documents, e.g., one group of documents might cover the treatment of *hypercholestorelemia* while the other group might deal with *hypertriglyceridemia*. When computing the results, an in-memory hash map is created that maps each variable substitution to a set of document ids. These groups are sorted in descending order by the number of documents in each group. Note that a document may have multiple substitutions, and hence, may appear in several groups. Hence, variable substitutions shared by many documents appear at the top of the list by default. Since the lists may become very long, we divided them into pages so that the user can jump to less frequent parts of the result list. In addition, the users may also sort the groups in ascending order (rare substitutions first). Our system visualizes a document group as a collapsible list item. A user’s click can uncollapse the list item to show all contained documents. Provenance information is used to explain why a document matches the query, i.e., the system displays the sentences in which a query’s pattern was matched. Provenance may be especially helpful when working with variable nodes.

*Hierarchical visualization.* Entities are arranged in taxonomies in many domains. Here, diseases, dosage forms, and methods are linked to MeSH (Medical Subject Heading) descriptors arranged in the MeSH taxonomy. The hierarchical visualization aims at showing document results in a hierarchical structure. For example, *hypercholestorelemia* and *hypertriglyceridemia* share the same superclass in MeSH, namely *hyperlipidemias*. All documents describing a treatment of *hypercholestorelemia* as well as *hypertriglyceridemia* are also matches to *hyperlipidemias*. On the backend side, we implemented an algorithm that works as follows:

1. Aggregate all documents by their variable substitution. Note that a document may have multiple substitutions, and hence, may appear in several groups.
2. Create an empty MeSH-tree structure.
3. Attach a set of documents to the corresponding tree position, i.e., the entity’s position in that tree.
4. Forward the number of documents to all predecessor nodes to update their document count.
5. Prune all nodes that do not have documents attached in their node or all successor nodes to bypass the need to show the whole MeSH taxonomy. Our service visualizes this hierarchical structure by several nested collapsible lists, e.g., *hyperlipidemias* forms a collapsible list. If a user’s click uncollapses this list, then the subclasses of *hyperlipidemias* are shown as collapsible lists as well. In this way, users can walk through the tree structure till they find document entries. These document entries are visualized in the same way as in our user interface when no variables are used.

## Retrieval evaluation

The following evaluations of our prototype are based on an older version (January 2021). In contrast to the content of the current version, which covers the complete Medline collection and COVID-19 pre-prints, the older version was focused on pharmaceutical users. Therefore, we selected a PubMed Medline subset that includes drug and excipient annotations. We annotated the whole Medline collection with our entity linking component, yielding 302 million annotations. Around six million documents included a drug or excipient annotation. Performing the extraction and cleaning workflow on around six million documents yielded nearly 270 million different extractions. Hence, the prototype version in January 2021 included about six million documents. In the following evaluation, we will thus call it prototype because we refer to the version of January 2021. The differences to the current system version were: (1) The content was smaller (not the complete NLM Medline and no pre-prints), (2) the entity vocabularies were older (older versions of MeSH and ChEMBL, and the entity types method, lab method and vaccine were missing), and (3) missing improvements in the user interface (improved document visualization, faster rendering, and faster loading).

Subsequently, we analyze our retrieval prototype concerning two research questions: *Do narrative query graphs offer a precise search for literature?* *And, do variable nodes provide useful entity-centric overviews of literature?* We performed three evaluations to answer these questions:

1. Two pharmaceutical experts created test sets to quantify the retrieval quality (100 abstracts and 50 full-text papers). Both experts are highly experienced in pharmaceutical literature search.
2. We performed interviews with eight pharmaceutical experts who search for literature in their daily research. Each expert was interviewed twice: Before testing our prototype to understand their information need and introducing our prototype. After testing our prototype, to collect feedback on a qualitative level, i.e., how they estimate our prototype’s usefulness.
3. Finally, all eight experts were asked to fill out a questionnaire. The central findings are reported in this paper.

### Retrieval evaluation

After having consulted the pharmaceutical experts, we decided to focus on the following typical information needs in the biomedical domain:

1. Drug-Disease treatments (*treats*) play a central role in the mediation of diseases.
2. Drugs might decrease the effect of other drugs and diseases (*decreases*).
3. Drug treatments might increase the expression of some substance or disease (*induces*).
4. Drug-Gene inhibitions (*inhibits*), i.e., drugs disturb the proper enzyme production of a gene.
5. Gene-Drug metabolisms (*metabolizes*), i.e., gene-produced enzymes metabolize the drug’s level by decreasing the drug’s concentration in an organism.

Narrative query graphs can specify the exact interactions a user is looking for. For each information need (I1-5), we built narrative query graphs with well-known entities from the pharmaceutical domain:

1. *Metformin treats Diabetes Mellitus (I1)*,
2. *Simvastatin decreases Cholesterol (I2)*,
3. *Simvastatin induces Rhabdomyolysis (I3)*,
4. *Metformin inhibits mtor (I4)*,
5. *CYP3A4 metabolizes Simvastatin AND Erythromycin inhibits CYP3A4 (I4/5)*, and
6. *CYP3A4 metabolizes Simvastatin AND Amiodarone inhibits CYP3A4 (I4/5)*.

**Table 7:** Expert evaluation of retrieval quality for narrative query graphs compared to PubMed and a MeSH-based search on PubMed. Two experts have annotated PubMed samples to estimate whether the information need was answered. Then, precision, recall, and F1-measure are computed for all systems

				PubMed	MeSH search			Narrative QG		
Query	#Hits	#Sample	#TP	Prec.	Prec.	Rec.	F1	Prec.	Rec.	F1
Q1	12.7K	25	19	0.76	0.82	0.47	0.60	**1**.**00**	0.42	0.59
Q2	5K	25	16	0.64	**0**.**73**	0.50	0.59	0.66	0.25	0.36
Q3	427	25	17	0.68	0.77	0.59	0.67	** 1.00**	0.35	0.52
Q4	726	25	16	0.64	**0**.**78**	0.44	0.56	0.71	0.31	0.43
Q5	397	25	6	0.24	–	–	–	**1**.**0**	0.17	0.29
Q6	372	25	5	0.20	–	–	–	**1**.**0**	0.20	0.33

- denotes no hits

For our evaluation, we wanted to measure our system’s precision and recall. The recall was of interest here because we already knew that information extraction (PathIE) could only extract statements between entities if mentioned in the same sentence. That is why we used the entities for each query to search for document candidates on PubMed, e.g., for Q1 we used *metformin diabetes mellitus* as the PubMed query. We kept only documents that were processed in our pipeline. Then, we took a random sample of 25 documents for each query. The experts manually read and annotated these sample documents’ abstracts concerning their information needs (true hits/false hits). Besides, we retrieved 50 full-text documents from PubMed Central (PMC) for a combined and very specialized information need (Q5 and Q6). The experts made their decision for PubMed documents by considering titles and abstracts, and for PMC documents, the full texts. We decided to select 25 as the sample size for each query because we had to obtain a manageable set of documents for our manual expert evaluation (in sum 100 abstracts and 50 full texts had to be evaluated). Subsequently, we considered these documents as ground truth to estimate the retrieval quality (precision, recall, and F1). Note that we did consider any ranking for the subsequent evaluation because matching narrative query graphs against document graphs is a binary decision: Either the information is contained or not. Ranking the results of such a ranking would require novel methods that were out of scope for this evaluation. However, we compared our retrieval to two baselines, (1) queries on PubMed and (2) queries on PubMed with suitable MeSH headings and subheadings.

*PubMed MeSH baseline* PubMed provides so-called MeSH terms for documents to assist users in their search process. MeSH is an expert-designed vocabulary comprising various biomedical concepts (around 26K different headings). These MeSH terms are assigned to PubMed documents by human annotators who carefully read a document and select suitable headings. Prime examples for these headings are annotated entities such as drugs, diseases, etc., and concepts such as study types, therapy types, and many more. In addition to headings, MeSH supports about 76 subheadings to precisely annotate how a MeSH descriptor is used within the document’s context. An example document might contain the subheading *drug therapy* attached to *simvastatin*. Hence, a human annotator decided that *simvastatin* is used in drug therapy within the document’s context. The National Library of Medicine (NLM) recommends subheadings for entity interactions such as treatments and adverse effects. In cooperation with our experts who read the NLM recommendations, we selected suitable headings and subheadings to precisely query PubMed concerning the respective entity interaction for our queries. We denote this baseline as *MeSH Search*.

*Results* The corresponding interaction and the retrieval quality (precision, recall, and F1-score) for each query are depicted in Table 7. The sample size and the number of positive hits in the sample (TP) are reported for each query. For instance, the sample size of Q2 was 25, and 16 documents were correct hits with regard to the corresponding information need. The subsequent reported precision, recall and F1 scores are based on the corresponding sample for each document.

The PubMed search was used to construct the ground truth, i.e., was used to retrieve the document lists from which the samples were drawn. That means that the PubMed search achieved a recall of 1.0 in all cases because all samples were subsets of the PubMed search results. The PubMed search yielded a precision of around 0.64 up to 0.76 for abstracts and 0.2 up to 0.24 for full texts. The PubMed MeSH search achieved a moderate precision of about 0.73-$-$0.82 and a recall of about 0.5 for PubMed titles and abstracts (Q1-Q4). Unfortunately, the relevant MeSH annotations were missing for all true-positive hits for Q5 and Q6 in PMC full texts. Hence, the PubMed MeSH search did not find any hits in PMC for Q5 and Q6. Narrative query graphs (Narrative QG) answered the information need with good precision: Q1 (*treats*) and Q3 (*induces*) were answered with a precision of 1.0 and a corresponding recall of 0.42 (Q1) and 0.47 (Q3). The minimum achieved precision was 0.66, and the recall differed between 0.17 and 0.42. Our prototype could answer Q5 and Q6 on PMC full texts: One correct match was returned for Q5 as well as for Q6, leading to a precision of 1.0.

#### Comparison to OpenIE

For our prototype, we used PathIE to extract the document graphs. For this comparison, we repeated the extraction on the benchmark documents by utilizing the Stanford CoreNLP OpenIE [25]. We selected the same relation vocabulary and cleaning rules. The results are listed in Table 8. By utilizing OpenIE we could not answer four out of six queries (Q1, Q3, Q5, Q6). A problematic example is the following sentence: *Metformin is the mainstay therapy for type 2 diabetes*. CoreNLP OpenIE extracted the following statement: (Metformin, is, mainstay therapy for type 2 diabetes). First, the object phrase contains more information than just the diabetes disease. Even if we would reduce the phrase to the pure disease *diabetes*, canonicalizing the verb phrase *is* to a *treats* relation would not be possible, simply because for all *is* verb phrases this decision would be wrong. Hence, CoreNLP OpenIE did not yield a suitable *treats* statement here to answer the query. In contrast, PathIE extracted a *treats* statement here because *therapy* was included in the relation vocabulary (a list of special words indicating a relation).

**Table 8:** Comparison between CoreNLP OpenIE and PathIE for narrative query graph retrieval (- no hits)

	OpenIE				PathIE		
	Prec.	Rec.	F1		Prec.	Rec.	F1
Q1	–	–	–		1.00	0.42	0.59
Q2	0.50	0.06	0.11		0.66	0.25	0.36
Q3	–	–	–		1.00	0.35	0.52
Q4	1.00	0.06	0.12		0.71	0.31	0.43
Q5	–	–	–		1.0	0.17	0.29
Q6	–	–	–		1.00	0.20	0.33

Although we achieved a precision of 1.0 for Q4 and 0.5 for Q2, in both cases the recall was at 0.06. In contrast, PathIE could answer all queries. For Q2 PathIE obtained a higher precision than OpenIE. For Q4 the precision was lower (0.71 instead of 1.0), but the recall was higher (0.31 instead of 0.11). In summary, PathIE was more suitable for our prototype because it could answer more queries and had a better F1 score for all queries.

### User interviews

The retrieval evaluation demonstrated that our system could achieve good precision when searching for specialized information needs. However, the following questions were: How does our prototype work for daily use cases? And, what are the prototype’s benefits and limitations in practice? Therefore, we performed two interviews with each of the eight pharmaceutical experts who search for literature in their daily work. All experts had a research background and worked either at a university or university hospital.

*First interview* We asked the participants to describe their literature search in the first interview. They shared two different scientific workflows that we had analyzed further: (1) Searching for literature in a familiar research area and (2) Searching for a new hypothesis which they might have heard in a talk or read in some paper. We performed think-aloud experiments to understand both scenarios. They shared their screen, showed us at least two different literature searches, and how they found relevant documents answering their information need. For scenario (1), most of them already knew suitable keywords, works, or journals. Hence, they quickly found relevant hits using precise keywords and sorting the results by their publication date. They already had a good overview of the literature and could hence answer their information need quickly. For scenario (2), they guessed keywords for the given hypothesis. They had to refine their search several times by varying keywords, adding more, or removing some. Then, they scanned titles and abstracts of documents looking for the given hypothesis. We believe that scenario (1) was recall-oriented: They did not want to miss important works. Scenario (2) seemed to be precision-oriented, i.e., they quickly wanted to check whether the hypothesis may be supported by literature. Subsequently, we gave them a short introduction to our prototype. We highlighted two features: The precision-oriented search and the usage of variable nodes to generate entity-centric literature overviews. We closed the first interview and gave them three weeks to use the prototype for their literature searches.

*Second interview* We asked them to share their thoughts about the prototype: What works well? What does not work well? What could be improved? First, they considered querying with narrative query graphs, especially with variable nodes, different and more complicated than keyword-based searches. Querying with variable nodes by writing *?X(Drug)* as a subject or an object was deemed too cryptic. They suggested that using *Drug*, *Disease*, etc. would be easier. Another point was that they were restricted to a fixed set of subjects and objects (all known entities in our prototype). For example, querying with pharmaceutical methods like *photomicrography* was not supported back then. Next, the interaction vocabulary was not intuitive for them. Sometimes they did not know which interaction would answer their information need. One expert suggested to introduce a hierarchical structure for the interactions, i.e., some general interactions like *interacts* that could be specified into *metabolizes* and *inhibits* if required. On a positive note, they appreciated the prototype’s precise search capability. They all agreed that they could find precise results more quickly using our prototype in comparison to other search engines. Besides, they appreciated the provenance information (why the document should be a match) to estimate if a document match answers their information need. They agreed that variable nodes in narrative query graphs offered completely new search possibilities, e.g., *In which dosage forms was Metformin used when treating diabetes?* Such a query could be translated into two fact patterns: (*Metformin*, *administered*, *?X(DosageForm)* and (*Metformin*, *treats*, *Diabetes Mellitus*). The most common administrations are done *orally* or via an *injection*. They agreed that such information might not be available in a specialized database like DrugBank. DrugBank covers different dosage forms for Metformin but not in combination with diabetes treatments. As queries get more complicated and detailed, such information can hardly be gathered in a single database. They stated that the *substitution-centric visualization* helps them to estimate which substitutions are relevant based on the number of supporting documents. Besides, they found the *hierarchical visualization* helpful when querying for diseases, e.g., searching for (*Metformin*, *treats*, *?X(Disease)*). Here, substitutions are shown in an hierarchical representation, e.g., *Metabolism Disorders*, *Glucose Disorders*, *Diabetes Mellitus*, *Diabetes Mellitus Type 1*, etc. They liked this visualization to get a drug’s overview of treated disease classes. All of them agreed that searches with variable nodes were helpful to get an entity-structured overview of the literature. Four experts stated that such an overview could help new researchers get better literature overviews in their fields.

### Questionnaire

We asked each domain expert to answer a questionnaire after completing the second interview. The essential findings and results are reported subsequently. First, we asked them to choose between precision and recall when searching for literature. Q1: *To which statement would you rather agree when you search for related work?* The answer options were (rephrased): A1a: *I would rather prefer a complete result list (recall). I do not want to miss anything.* A2a: *I would rather prefer precise results (precision) and accept missing documents*. Six of eight experts preferred recall, and the remaining two preferred precision. We asked a similar question for the second scenario (hypothesis). Again, we let them select between precision and recall (A1a and A1b). Seven of eight preferred precision, and one preferred recall when searching for a hypothesis. Then, we asked Q3: *To which statement would you rather agree for the vast majority of your searches?* Again, seven of eight domain experts preferred precise hits over complete result lists. The remaining one preferred recall. The next block of questions was about individual searching experiences with our prototype (called prototype in the Questionnaire): different statements were rated on a Likert scale ranging from 1 (disagreement) to 5 (full agreement). The results are reported in Table 9. They agreed that the prototype allows to formulate precise questions (4.0 mean rating), and the formulation of questions was understandable (4.0). Besides, provenance information was beneficial for our users (5.0). They could well imagine using our prototype in their literature research (3.9) and searching for a hypothesis (3.4). Still, users were reluctant to actually switch to our prototype for related work searches (2.8). Finally, the result visualization of narrative query graphs with variables was considered helpful (4.5).

**Table 9:** Questionnaire results: eight participants were asked to rate the following statements about our prototype on a Likert scale ranging from 1 (disagreement) to 5 (agreement). The mean ratings are reported

Statement about the prototype	Mean
The prototype allows me to formulate precise questions by specifically expressing the interactions between search terms	4.0
The formulation of questions in the prototype is understandable for me	4.0
The displayed text passage from the document (Provenance) is helpful for me to understand why a document matches my search query	5.0
The prototype provides precise results for my questions (I quickly find a relevant match)	3.5
Basically, grouping results is helpful for me when searching for variable nodes	4.5
When searching for related work, I would prefer the prototype to a search using classic search tools (cf. PubPharm, PubMed, etc)	2.8
When searching for or verifying a hypothesis, I would prefer the prototype to a search using classic search tools (cf. PubPharm, PubMed, etc).	3.4
I could imagine using the prototype in my literature research	3.9

### Performance analysis

We measured the performance of our prototype and database on a server, having two Intel Xeon E5-2687W (@3,1 GHz, eight cores, 16 threads), 377 GB of DDR3 main memory, and SDDs as primary storage. Back in January 2021, the preprocessing took around one week for our six million documents (titles and abstracts). We have incrementally improved the performance and can now process the complete Medline collection (34 M documents) in one week. We randomly generated 10 k queries asking for one, two, and three interactions. We measured the query execution time on a single thread (on the January 2021 version). Queries that are not expanded via an ontology took in average 21.9 ms (1-fact) / 52 ms (2-facts) / 51.7 ms (3-facts). Queries that are expanded via an ontology took in average 54.9 ms (1-fact) / 158.9 ms (2-facts) / 158.2 ms (3-facts). However, the query time heavily depends on the interaction (selectivity) and how many subclasses are involved. In summary, our system can retrieve documents within a quick response time for the vast majority of searches.

## Discussion

In close cooperation with domain experts using the PubMed corpus, our evaluation shows that overall document retrieval can indeed decisively profit from graph-based querying. The expert evaluation demonstrates that our system achieves moderate up to good precision for highly specialized information needs in the pharmaceutical domain. Although the precision is high, our system has only a moderate recall. Moreover, we compared our system to manually curated annotations (MeSH and MeSH subheadings), which are a unique feature of PubMed. Most digital libraries may support keywords and tags for documents but rarely support how these keywords, and primarily, how entities are used within the document’s context. Therefore, we developed a document retrieval system with a precision comparable to manual metadata curation but without the need for manual curation of documents.

The user study and questionnaire reveal a strong agreement for our service’s usefulness in practice. In summary, the user interface must be intuitive to support querying with narrative query graphs. Further enhancements are necessary to explain the interaction vocabulary to the user. We appreciate the idea of hierarchical interactions, i.e., showing a few basic interactions that can be specified for more specialized needs. Especially the search with variable nodes in detailed narrative query graphs offers a new access path to the literature. The questionnaire showed that seven of eight experts agreed that the vast majority of their searches are precision-oriented. Next, they agreed that they prefer our service over established search engines for precision-oriented searches. The verification of hypotheses seems to be a possible application because precise hits are preferred here. We believe that our service should not replace classical search engines because there are many recall-oriented tasks like related work searches. The recall will always be a problem by design when building upon error-prone natural language processing techniques and restricting extractions to sentence levels. Although the results seem promising, there are still problems to be solved in the future, e.g., we can still improve the extraction and the user interface.

### Technical challenges

We faced five major technical challenges when realizing narrative query graph retrieval:

*Retrieval with graphs* Graph-based retrieval of literature requires representing texts differently. We decided to extract statements from text, compute an inverted index, and then compute queries against that index. An alternative could be a retrieval with the latest language models that may match query graphs on-the-fly against texts.

*Data storage and query processing* The processing of queries requires performing an expensive graph-pattern matching. Here we built upon relational databases to store all data within one place. But alternatives like performing queries on a graph database and retrieving Provenance from a different source could be relevant.

*Query formulation* Formulating information needs as query graphs was unfamiliar and thus challenging for users. Easy-to-use interfaces must be developed and integrated here. An extension could be to formulate natural language questions that are automatically translated to query graphs.

*Result list handling* Transferring result lists between backend and frontend can be similar to keyword-based retrieval systems. But if variables came into play and result lists became nested, a new way of handling that lists was required. Either we must recompute the query for certain list parts or design a suitable caching and streaming architecture.

*Querying with variables* Searches with variables required novel methods to transfer and visualize the result lists: On the one hand, the visualization must face a large amount of data in real-time. On the other hand, interfaces should be fast and responsive.

Our discovery system tackled all challenges by finding solutions that worked in practice and delivered a suitable quality.

### Generalizability

Knowledge in the biomedical domain is often entity-centric, e.g., clinical studies involving certain target groups, drug testing, treatments and therapies, method investigations, and much more. Existing thesauri and distinguishing relations between certain entities were essential for realizing access via narrative query graphs. The generalizability of this research is in this way limited to entity-centric domains. In political sciences, information needs may be based upon some school of thought. For example, when searching for statements of that school, special keywords and framing are essential to formulate the actual query. Breaking down such information need to a *simple* entity-interaction pattern does not seem possible.

On the one hand, we have already seen methods suitable for our extraction workflow, and hence, our discovery system would not work well for political sciences if suitable vocabularies were missing [19]. Canonicalizing different predicates to precise relations here is even more challenging. In biomedicine, a drug and a disease might roughly stand in two relations: Either the drug treats the disease or induces the disease. When thinking about possible relations between persons, we are likely to face a high number of possible ones. But on the other hand, although having these restrictions, we have elaborated on the benefits of narrative information access for political sciences [20]. For example, listing people involved in a certain decision, or structuring the literature into action categories, e.g., tackling climate change, could be answered by realizing a similar access path to that domain.

## Outlook

In addition to steady improvements in the user interface, entity vocabularies, and content updates, we give an outlook on the latest developments of our service.

**Fig. 5:**
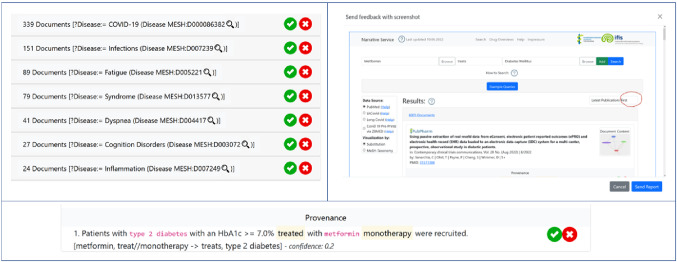
Feedback options of our service: on the left upper corner, users can rate substitution groups when searching with variables. On the right upper corner, users can create a screenshot, mark something in that, write a short text, and send it to us. At the bottom, users can rate Provenance entries (if the extraction was suitable)

**Fig. 6:**
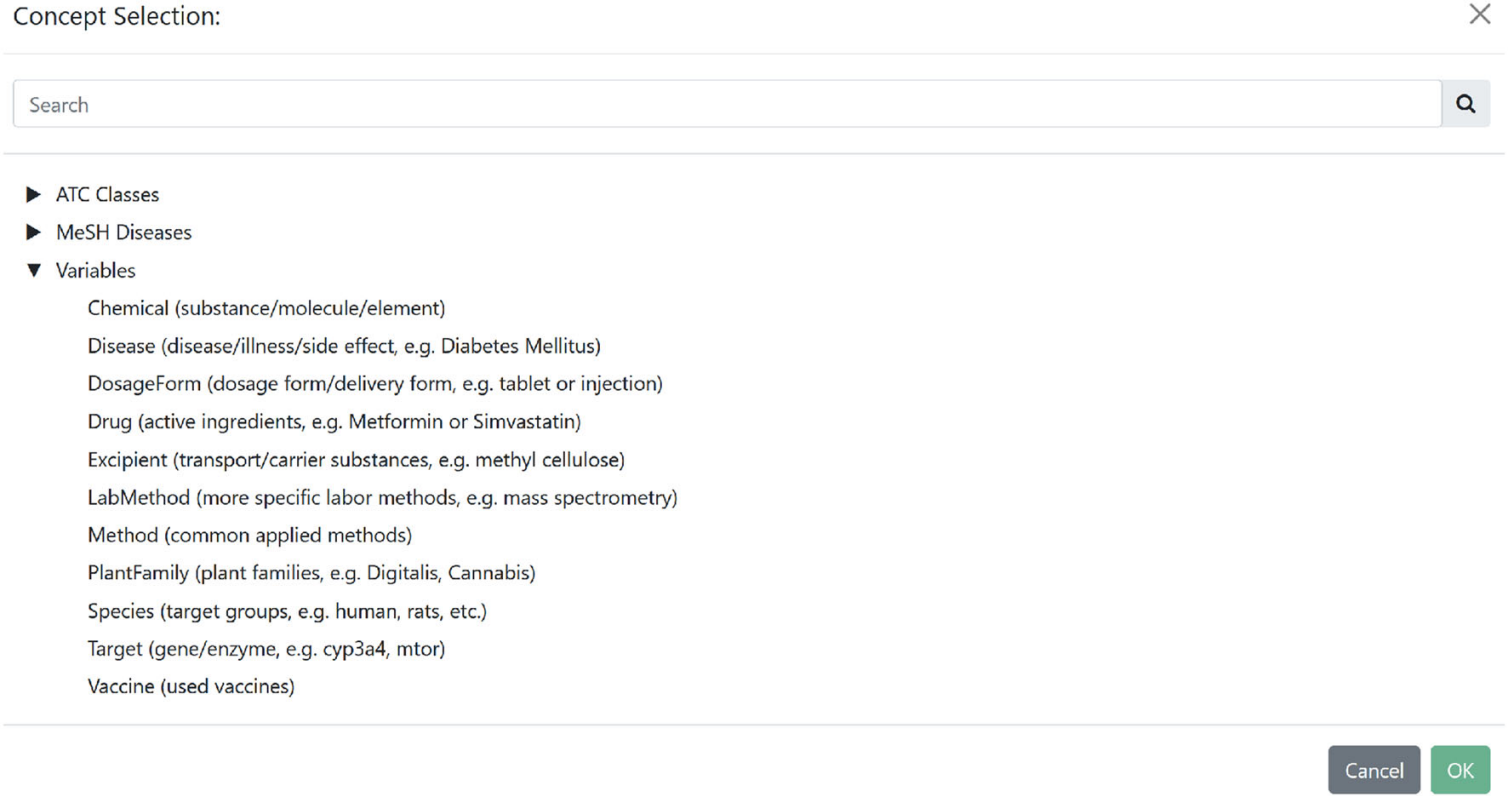
Concept selection: this view allows users to precisely select their concept (entity/class) or a typed variable (variable of type drug, etc.) in searches. The view has a search field, so the tree-based visualization can be searched in real-time

### Feedback

This work’s qualitative and quantitative evaluations show that users can benefit from narrative query graphs in practice. However, we continue the evaluation of our service. For instance, two preliminary evaluations are described in  [20]: In joint work with the specialized information services for political sciences (Pollux), we analyzed how well the service can assist research questions in political sciences. In cooperation with ZB MED (infrastructure and research center for information and data in the life sciences) and Robert Koch-Institute in Germany (leading public health institute in Germany), we evaluate how well the service is suitable to answer COVID-9-related questions.

For our daily users, we integrated three feedback options into our system: (1) Users can automatically create a screenshot of our system, mark something in that and write a short text. Then the data is sent automatically to our service. (2) Users can rate visualized substitution groups when searching with variables. Users who explore substitution lists can directly rate if this group is sensible concerning the query. (3) Users can rate Provenance information, i.e., whether the extraction is suitable to answer the query. The options are shown in Fig. 5. All this feedback is stored in our service, and we will further use it to improve the system and extraction methods.

### Concept selection

In our study, we learned that selecting the correct concept (entity/class or variable) can sometimes be challenging. On the one hand, users might not know the correct term for a given entity, e.g., users searched for *diabetes* instead of the correct entity term *Diabetes Mellitus*. On the other hand, users did not know which overviews could be generated, i.e., which variable types we allowed. To deal with this problem, we introduced the so-called Concept Selection View. A screenshot is shown in Fig. 6. Here users can enter a term, and a list of matching concepts (entities/classes/variables) is shown. In addition, we integrated a list of allowed variable types and classes from MeSH and ATC. This view extends the autocompletion function by showing a tree-based visualization of concepts, i.e., we utilized ontologies like MeSH and ATC (a drug taxonomy) to show the different concepts and their superclass/subclass relationships. If a user selects a concept here, the concept will be copied to the query builder. Users can access the concept selection by clicking on the *Browse* button either in the subject or object of the query builder.

**Fig. 7:**
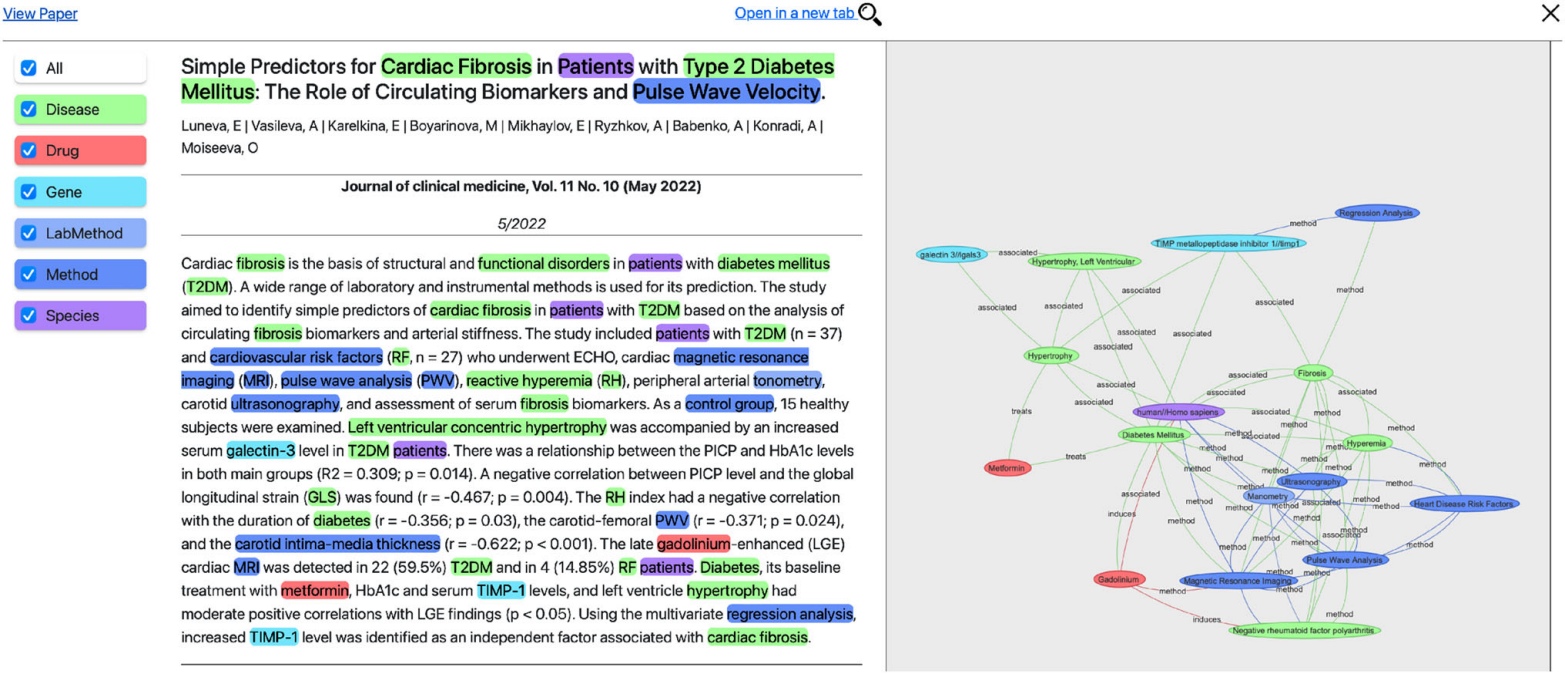
Document graph visualization: a document’s abstract and identified entities are highlighted on the left side. On the right side, interactions between entities (statements) are visualized as a directed-edge labeled and colored graph. Different colors depict different entity types (drugs, diseases, etc)

### Document graphs

Our service already allowed the visualization of Provenance information, i.e., the service explains why a document should match a query graph. However, we found it to be useful to allow users to explore our actual document graph representation as well. Therefore, we integrated a Document Graph View. A screenshot is shown in Fig. 7. This view has two components: On the left side, the document’s text plus metadata (authors, journals, etc.) is shown. Here we highlight detected entities in the title and abstract in a certain color (the color denotes the entity type). Users have the option to select or deselect certain entity types to focus the visualization on their needs. On the right side, the actual document graph is visualized as a directed-edge labeled and colored graph. Colors again denote the entity types. The graph is interactive, and users may move nodes or edges. If two entities are connected via several predicates, we only visualize a single edge and concatenate the predicate labels, e.g., *associated and treats*.

### Drug overviews

**Fig. 8:**
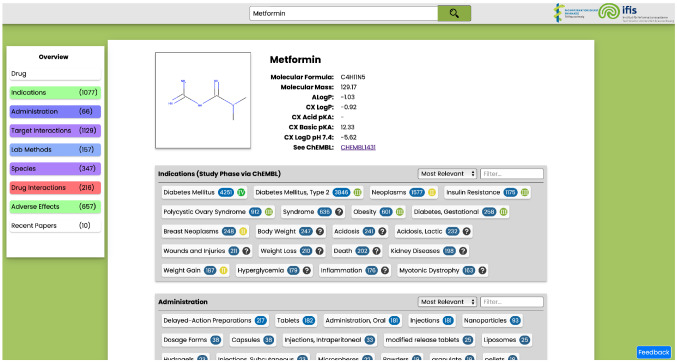
Drug overviews: users enter a drug name, and then an overview of the drug is generated. Therefore we combine information from our service as well as from curated and specialized databases. A user’s click on an entity will then invoke a search in our discovery system

Our latest extension is the so-called *Drug Overviews*. A screenshot is shown in Fig. 8. Users have to enter a drug name, and the corresponding overview is generated for them. Therefore, we combine information from our service as well as from curated and specialized databases. On the one hand, we show curated information about the drug like the molecular mass, pKA values, etc. To retrieve the curated information, we utilized the official API of the ChEMBL database [27]. On the other hand, a set of pre-defined narrative query graphs is used to show extractions from the literature. In cooperation with domain experts again, we created these query graphs for different purposes: Showing known indications (treatments) of the drug, showing how the drug is administered (tablet, injection, etc.), the interacting targets (enzymes/gene systems) and more. Then the corresponding results, i.e., the entity groups, are shown in list views. Each entry consists of two components: the entity’s name and the number of supporting documents for the given relationship between the searched drug and this entity. Users who click on an entity will be forwarded to our retrieval service, and the corresponding relationship is searched automatically. Another thing to mention are indications: Here, we combine extractions from the literature with information about clinical phases from ChEBML [27]. Suppose a drug-disease-indication is verified via a clinical trial. In that case, the corresponding phase of the trial is visualized as a roman letter in its entry. Users who click on the trial phase will be forwarded to the corresponding ChEBML entry.

The difference with existing curated databases is that we can generate these overviews even for the latest drugs. And moreover, we can show associations that may have been reported in the literature but have not been curated in a database. For instance, a drug administration as a nanoparticle might not have worked out in practice. It likely will not appear in a curated database but is shown in our overview. In summary, these overviews allow thus to quickly retrieve information about a drug, even if the drug has not been researched thoroughly.

## Conclusion

Entity-based information access catering even for complex information needs is a central necessity in today’s scientific knowledge discovery. But while structured information sources such as knowledge graphs offer *high query expressiveness* by graph-based query languages, scientific document retrieval is severely lagging behind. The reason is that graph-based query languages allow to describe the desired characteristics of and interactions between entities in sufficient detail. In contrast, document retrieval is usually limited to simple keyword queries. Yet unlike knowledge graphs, scientific document collections offer *contextualized knowledge*, where entities, their specific characteristics, and their interactions are connected as part of a coherent argumentation and thus offer a clear advantage [14, 15]. The research presented in this paper offers a novel workflow to bridge the worlds of structured and unstructured scientific information by performing graph-based querying against scientific document collections. Implementing such an access path to a digital library comes with costs for designing extraction workflows and maintaining the actual discovery system. However, nearly unsupervised extraction workflows might be a compromise here: They bypass training data for the extraction but suffer in quality and require suitable vocabularies. If a digital library decides to go that way, novel applications such as the query graph retrieval system, searches with variables, graph visualizations of documents, or overviews of certain entities (here drugs) are not too far-fetched. But as our current workflow is clearly precision-oriented, we plan to improve the recall without having to broaden the scope of queries in future work.

**Supplementary information** The code of the extraction toolbox can found in our GitHub repository: https://github.com/HermannKroll/KGExtractionToolbox. An archived version of our toolbox can be found in the Software Heritage project: https://archive.softwareheritage.org/swh:1:dir:67c17339a5c800ddb50cb36bda598fb96a200856.

## Footnotes

1 https://github.com/HermannKroll/KGExtractionToolbox.

2 https://meshb.nlm.nih.gov.

3 https://www.ncbi.nlm.nih.gov/research/pubtator/.

4 http://www.narrative.pubpharm.de.
